# Reduction of dopant ions and enhancement of magnetic properties by UV irradiation in Ce-doped TiO_2_

**DOI:** 10.1038/s41598-021-87115-z

**Published:** 2021-04-07

**Authors:** Tai-Sing Wu, Leng-You Syu, Bi-Hsuan Lin, Shih-Chang Weng, Horng-Tay Jeng, Yu-Shan Huang, Yun-Liang Soo

**Affiliations:** 1 National Synchrotron Radiation Research Center, Hsinchu, Taiwan; 2 Department of Physics, National Tsing Hua University, Hsinchu, Taiwan; 3 Institute of Physics, Academia Sinica, Taipei, Taiwan

**Keywords:** Ferromagnetism, Magnetic properties and materials, Semiconductors, Structure of solids and liquids

## Abstract

We report the experimental observation of and theoretical explanation for the reduction of dopant ions and enhancement of magnetic properties in Ce-doped TiO_2_ diluted magnetic semiconductors from UV-light irradiation. Substantial increase in Ce^3+^ concentration and creation of oxygen vacancy defects in the sample due to UV-light irradiation was observed by X-ray and optical methods. Magnetic measurements demonstrate a combination of paramagnetism and ferromagnetism up to room temperatures in all samples. The magnetization of both paramagnetic and ferromagnetic components was observed to be dramatically enhanced in the irradiated sample. First-principle theoretical calculations show that valence holes created by UV irradiation can substantially lower the formation energy of oxygen vacancies. While the electron spin densities for defect states near oxygen vacancies in pure TiO_2_ are in antiferromagnetic orientation, they are in ferromagnetic orientations in Ce-doped TiO_2_. Therefore, the ferromagnetically-oriented spin densities near oxygen vacancies created by UV irradiation are the most probable cause for the experimentally observed enhancement of magnetism in the irradiated Ce-doped TiO_2_.

## Introduction

Titanium-dioxide-based diluted magnetic semiconductors (DMS) have been widely considered as promising ferromagnetic material possibly useful for spintronic applications. The structures, electronic states, and magnetic properties in transitional metal-doped TiO_2_ have been previously studied^1–4^. However, despite reports of successful materials synthesis and favorable magnetic properties, questions remain regarding the origin of the materials’ magnetism and consequently their applicability in spintronics^5^. On the other hand, it is possible that the intrinsically non-magnetic CeO_2_ may undergo magnetic phase transition when oxygen vacancy defects (V_O_) are introduced to the material. As reported in a recent paper, UV-irradiated CeO_2_ indeed demonstrates enhanced room-temperature ferromagnetism^6^. UV irradiation is thought to generate oxygen vacancies, converting a substantial fraction of constituent Ce^4+^ into subvalent Ce^3+^ and thus enhancing ferromagnetic ordering in the ceria sample. It is therefore conceivable that a new species of TiO_2_-based DMS may be obtained by doping Ce into titanium dioxide hosts followed by UV irradiation.

In the present work, we have used rare-earth element Ce as a replacement for transition metal ions in the synthesis of TiO_2_-based DMS. The Ce-doped ceria samples were irradiated with UV light to progressively increase the concentration of subvalent Ce^3+^ ions. X-ray absorption near-edge structure (XANES) and extended X-ray absorption fine structure (EXAFS) techniques were employed to monitor the changes in Ce^3+^ concentration and the number of oxygen vacancies surrounding Ce, respectively. It has been demonstrated recently that the Ce-L-edge XANES data are especially useful for studying the magnetic and catalytic properties in doped CeO_2_ nanoparticles^7,8^. The variation of band gaps as a result of UV irradiation was also determined by UV–Vis diffuse reflectance measurements. Photoluminescence (PL) spectra were used to investigate the change of defect states due to UV irradiation. Finally, the magnetic property changes were experimentally observed by using a superconducting quantum interference device (SQUID) and theoretically understood by performing first-principle density functional theory (DFT) calculations.

## Experimental

**Figure 1:**
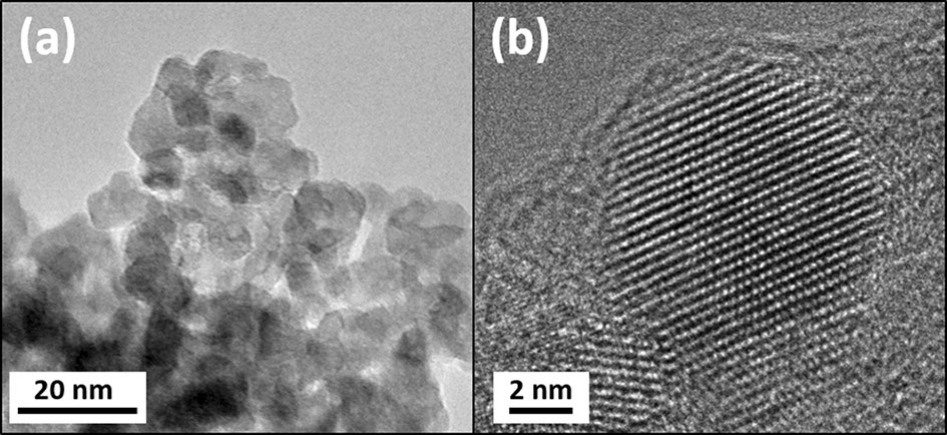
(**a**) Medium- and (**b**) high-resolution TEM images of the as-made Ce-doped TiO_2_ samples.

Nanocrystal samples of Ce-doped TiO_2_ were prepared using a sol–gel method^9^. As shown in Supplementary Fig. S1 online, the synchrotron-based X-ray powder diffraction pattern shows exclusively anatase TiO_2_ Bragg peaks; no cerium oxide phase was detected. The crystallite size was determined by the Scherrer equation to be 6.4 nm. The Ce concentration was measured by inductively coupled plasma mass spectrometry (ICPMS) to be 0.98 at%. As shown in Fig. 1, the TEM images demonstrate that the Ce-doped TiO_2_ samples have rather good crystallinity and the size of the nanoparticles is consistent with that obtained from the Scherrer equation. To study the UV-light irradiation effects, Ce-doped TiO_2_ samples were irradiated by UV-light with various irradiation time durations in ambient condition. A handheld UVC lamp (6 W, UVGL-58, UVP) was used with an average power of 2.5 mw/cm^2^ on the sample. After UV-light irradiation, Ce L_3_-edge and Ti K-edge XANES and EXAFS were performed to monitor the evolution of valances and local structures surrounding Ce and Ti atoms, respectively. All XANES and EXAFS measurements were performed in transmission mode at beamline BL07A of the Taiwan Light Source (TLS) at National Synchrotron Radiation Research Center (NSRRC). To monitor the electronic structural changes due to UV-light irradiation, the UV–Vis diffuse reflectance measurements were carried out using a spectrophotometer (UV-4150, Hitachi). Photoluminescence (PL) spectra of the as-made and irradiated samples were also measured using an excitation source of wavelength 325 nm to investigate the variation of defect states due to UV-light irradiation. Variations of the sample’s magnetic properties due to UV-light irradiation were monitored by M–H and M–T measurements using a superconducting quantum interference device (SQUID).

## Results and discussion

**Figure 2:**
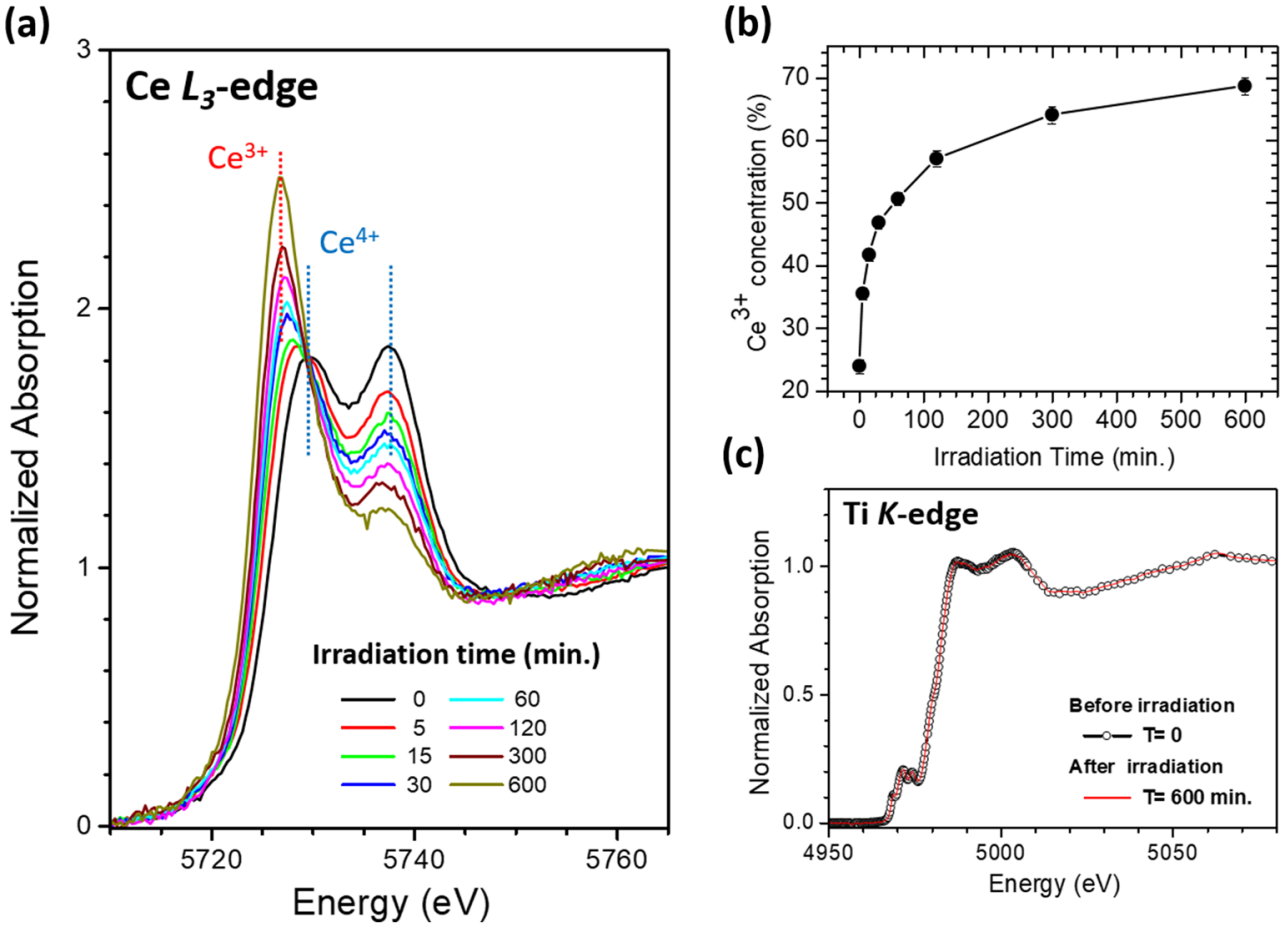
(**a**) Ce L_3_-edge XANES of UV-light irradiated sample with various irradiation times. (**b**) Plot of Ce^3+^ concentration vs. irradiation time. (**c**) Ti K-edge XANES of sample before and after 600 min of UV-light irradiation.

The Ce XANES of samples with different UV-light irradiation time durations are shown in Fig. 2a. The peak ascribed to Ce^3+^ increases with the irradiation time, representing reduction of Ce^4+^ as a result of UV irradiation. To extract Ce^3+^ concentration from XANES data, the spectra were curve-fitted with an arctangent function to simulate the edge jump and Gaussian functions for peak features. Details of the curve-fitting method have been previously reported^10^. It’s worth noting that application of Ce-L-edge XANES on the studies of magnetism and photocatalysis in doped CeO_2_ has also been reported recently^7,8^. As shown in Fig. 2b, after 600 min of UV-light irradiation, the Ce^3+^ concentration increased from 23.9% and saturated at 68.6%. Figure 2c shows the Ti XANES of the samples before and after UV-light irradiation. Notably, despite the dramatic changes observed in Ce XANES, the Ti XANES showed the same spectrum even after 600 min of UV-light irradiation, representing an unchanged or at most marginally affected Ti valence state.

**Figure 3:**
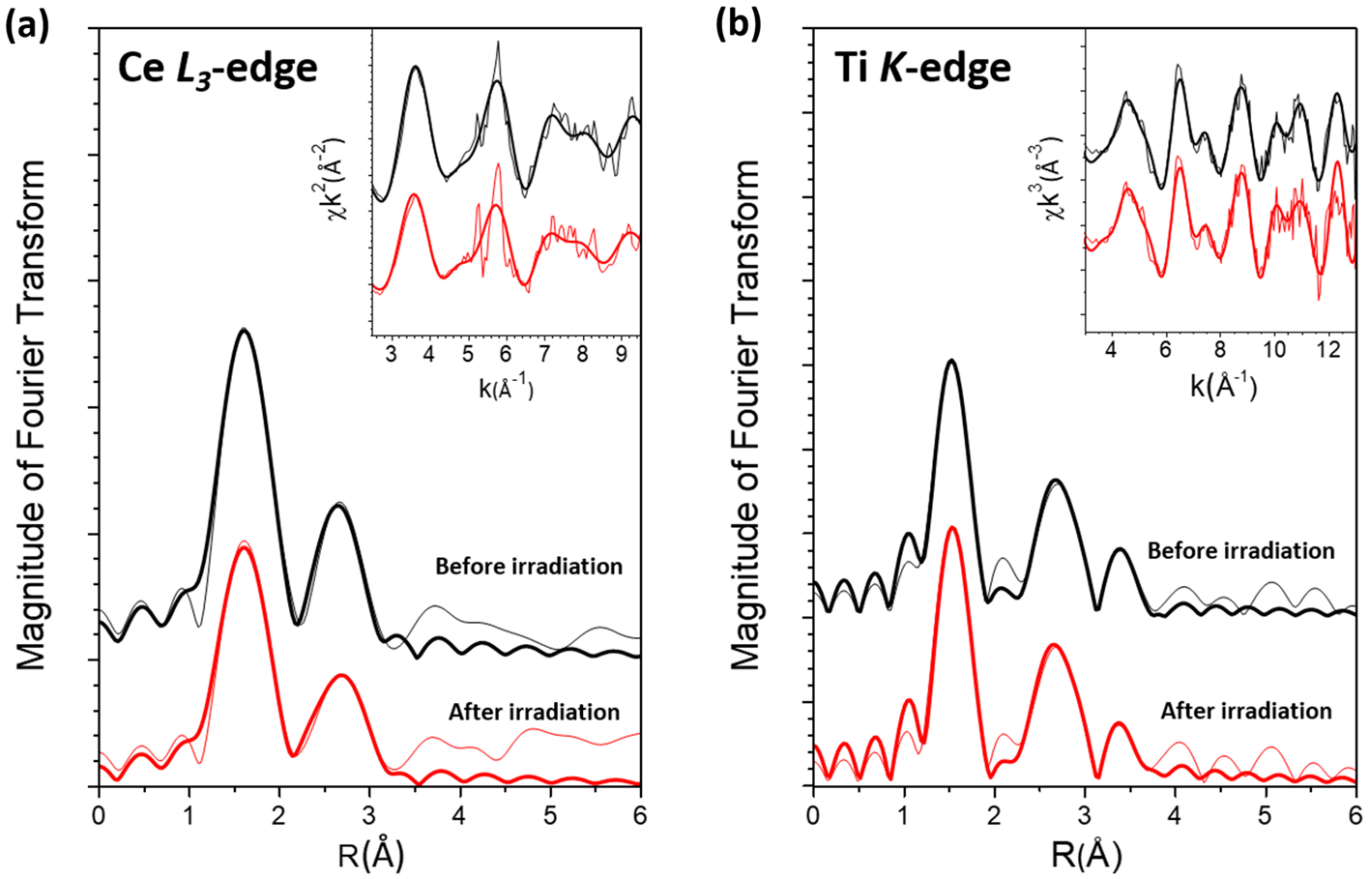
(**a**) Ce L_3_-edge and (**b**) Ti K-edge EXAFS data for the sample before and after 600 min of UV-light irradiation. Fine lines: experimental; Coarse lines: curve fitting. Curves have been shifted vertically for the sake of clarity**.**

The Ce L_3_-edge and Ti K-edge EXAFS analyses were also performed using the *ARTEMIS* package^11^ to probe the local structural variations due to UV-light irradiation surrounding the Ce dopant atoms and Ti constituent atoms, respectively, in Ce-doped TiO_2_. As shown in Fig. 3a and Supplementary Table S1 online, the as-made sample has one O shell at 2.13 ± 0.01 Å and a Ti shell at 3.18 ± 0.02 Å from the Ce central atom, with coordination numbers 5.7 ± 0.5 and 3.4 ± 0.3, respectively. The Ce–O bond length of 2.13 Å observed in the as-made sample is substantially shorter than that of 2.34 Å in the fluorite ceria structure. However, except for longer interatomic distances due to larger ionic radius of Ce^4+^ (87 pm) compared to that of Ti^4+^ (61 pm), the local structural parameters for Ce in the as-made sample resemble those for Ti in TiO_2_, in which 6 O atoms and 4 Ti atoms are located at 1.95 Å and 3.04 Å from the central Ti central atom, respectively. We therefore conclude that the Ce dopant atoms in the as-made Ce-doped TiO_2_ sample are most likely substitutionally incorporated into the host matrix, occupying Ti sites in TiO_2_. After 600 min of UV-light irradiation, as listed in Supplementary Table S1 online, the coordination number of the (O) nearest neighboring shell is reduced from 5.7 to 3.8, representing the formation of O vacancy surrounding the Ce dopant atoms as a result of UV irradiation. This is consistent with the Ce XANES results that Ce^4+^ is reduced to Ce^3+^ due to UV-light-induced Ce–O bond breakage in the irradiated sample.

In contrast to the Ce local structure which can be substantially changed by UV irradiation, the local structure surrounding Ti atoms appears to remain largely intact after 600 min of UV-light irradiation. As shown in Fig. 3b, the Fourier transform of the Ti EXAFS χ-function for the irradiated sample generally resembles that for the as-made sample. Within fitting uncertainties, the final values of the local structural parameters obtained from curve-fittings for the irradiated samples are also unchanged from those for the as-made samples (Supplementary Table S2 online). Therefore, our EXAFS analysis is consistent with the XANES results that UV irradiation changes the local chemical environment surrounding Ce dopant atoms without inducing substantial variation to that surrounding Ti constituent atoms in the TiO_2_ host.

**Figure 4:**
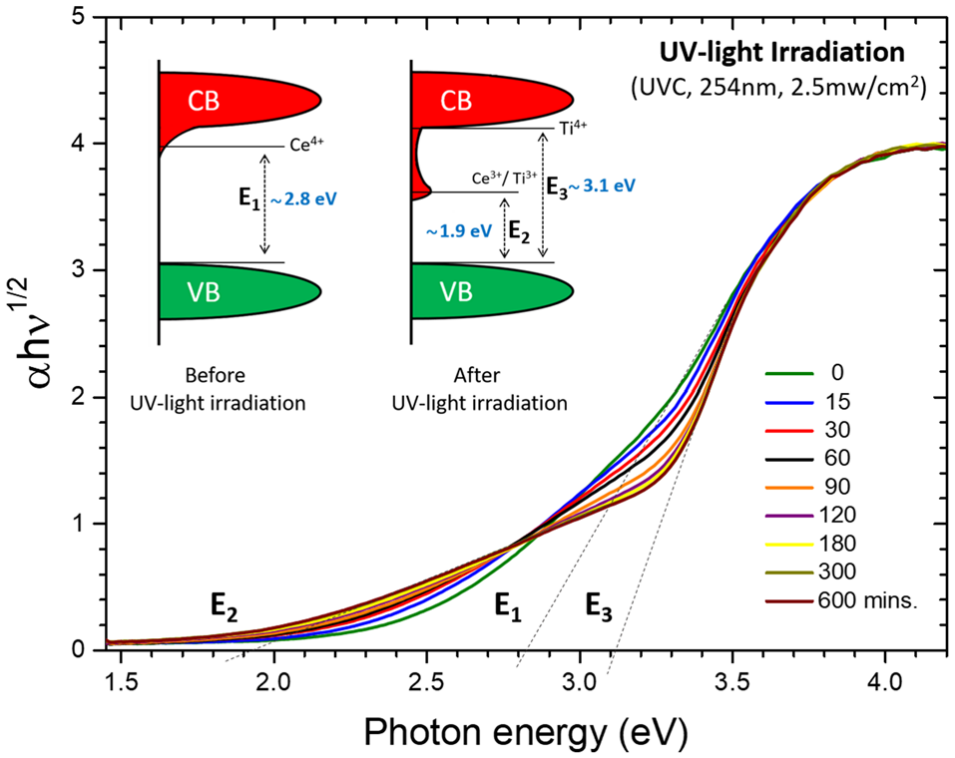
Plot of ${(\alpha hv)}^{1/2} \mathrm{vs.} hv$ deduced from the UV–Vis diffuse reflectance spectra of UV-light irradiated sample with various irradiation times.

The band gap values of the as-made sample and the samples irradiated for different time durations were extracted from the UV–Vis diffuse reflectance data using the Kubelka–Munk function^12^ and Tauc’s plots^13^ as shown in Fig. 4. The 2.8 eV (E_1_) band gap value for the as-made sample is smaller than the reported 3.2 eV indirect band gap of anatase TiO_2_^14^. According to the DFT calculations published by Albuquerque et al.^15^, the Ce^4+^ (4*f*^0^) band for Ce dopant atoms deep in bulk TiO_2_ completely overlaps with the Ti 3d band while those for the subsurface and surface Ce atoms are just below and well below the Ti 3d states, respectively. The observed band-gap narrowing is ascribed to the presence of a Ce^4+^ impurity band in the energy gap that extends past the conduction band minimum of TiO_2_ due to Ce doping.

For the UV-light irradiated sample, it was observed that a band-tailing effect progressively emerged with increase in irradiation time. As shown in Fig. 4, the initially smooth ${(\alpha hv)}^{1/2} \mathrm{vs. }hv$ curve is gradually lowered in the region from 2.8 to 3.4 eV and raised in that from 1.9 to 2.8 eV due to UV-light irradiation. Consequently, the energy band gap E_1_ for the as-made sample gradually split into a smaller gap E_2_ and a larger gap E_3_ as the irradiation time increases. As revealed by previous DFT calculations and X-ray measurements, the Ti^3+^ and Ce^3+^ defect states spawned by UV-light irradiation formed conduction band tail states that extend into the energy band gap and decreases the band gap value^15,16^. After 600 min of UV irradiation, the band gap value decreased to 1.9 eV (E_2_). As a consequence of the Ce^4+^ to Ce^3+^ conversion, an additional higher band gap of 3.1 eV (E_3_) was also observed, which can be attributed to the valance band to Ti^4+^ transition.

**Figure 5:**
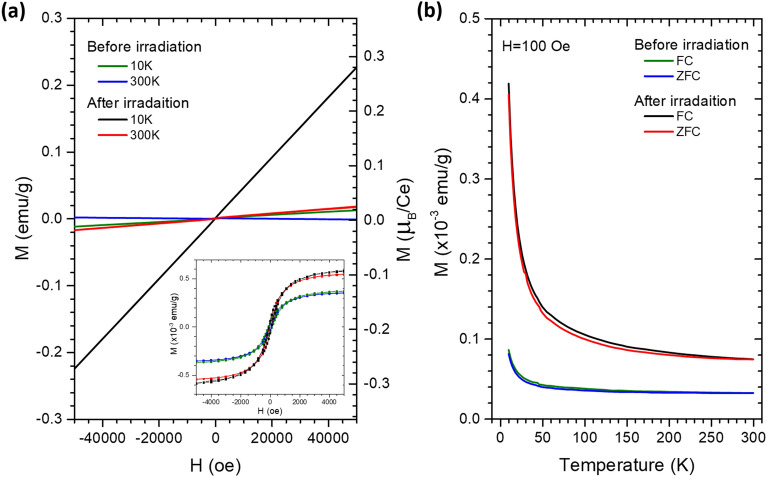
(**a**) M-H curves and (**b**) FC/ZFC curves of Ce-doped TiO_2_ sample before and after 600 min of UV-light irradiation. The inset of (**a**) shows the ferromagnetic component of each curve obtained by subtracting the paramagnetic background from the original curve.

The effects of UV-light irradiation on the sample’s magnetic properties were monitored by SQUID measurements. As revealed in Fig. 5, the M–H curve of the as-made sample exhibits a combination of ferromagnetism and paramagnetism up to room temperatures. After UV-light irradiation, the magnetization in the sample was substantially increased compared to that in the as-made sample. The field cooled (FC) and zero field cooled (ZFC) magnetization measurements under an applied field of 200 Oe also show enhanced magnetization due to UV-light irradiation up to the temperature of 300 K.

## Theoretical calculations

To explain the observed enhancement of magnetic properties of Ce-doped TiO_2_ due to UV-light irradiation, spin-polarized DFT calculations using VASP codes were employed to estimate the formation energies of O vacancy defects on different sites^17^. The projector augmented-wave (PAW) method and the generalized gradient approximation (GGA) within the parameterization of Perdew–Burke–Ernzerhof (PBE) were used in the calculation^18^. The Ce (5s^2^5p^6^6s^2^4f^1^5d^1^), Ti (3p^6^4s^2^3d^2^) and O (2s^2^2p^4^) electrons were treated as valence electrons and their state functions were expanded on a plane-wave basis with a kinetic energy cutoff of 500 eV. A GGA + U method was used for the correction of on-site Coulomb interactions with U = 4 eV applied to both the Ce 4f.- and Ti 3*d*-states. As reported previously, an accumulation of positive charge was observed on the TiO_2_ surface during UV-light irradiation^19^. Different numbers of valence electrons were removed from the supercell in our calculation to mimic the creation of valence holes during UV-light irradiation. The initial structural model used for the DFT calculation was constructed from a 2 × 2 × 2 supercell of anatase TiO_2_. To construct the structural model for Ce-doped TiO_2_, one Ti atom was replaced by a Ce atom in the 96-atom supercell, resulting in a Ti_0.97_Ce_0.03_O_2_ stoichiometry (3.1% Ce).

**Figure 6:**
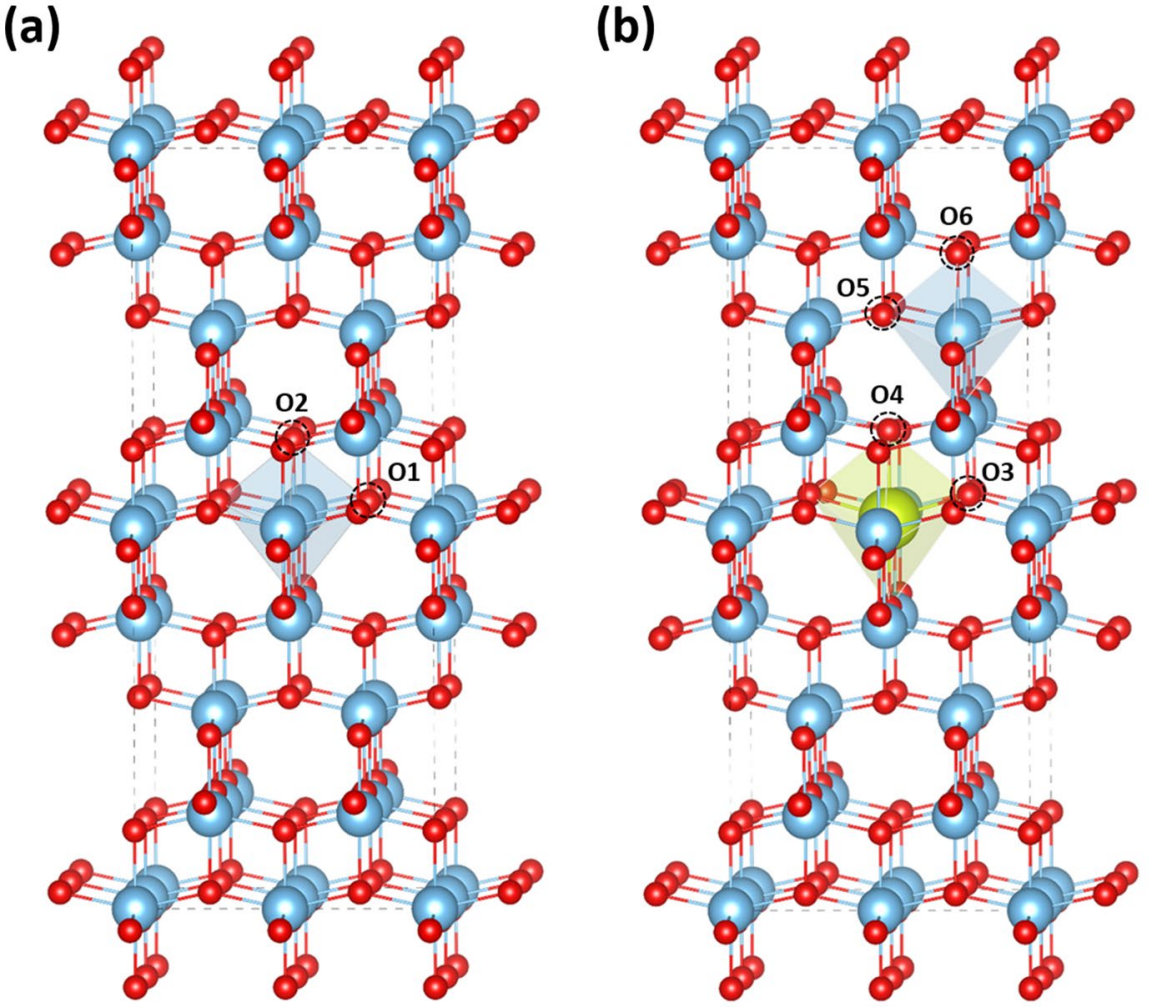
Schematic view of undoped and Ce-doped anatase TiO_2_. (**a**) The possible O vacancy sites V_O1_ and V_O2_ in a [TiO_6_] octahedron of the undoped TiO_2_. Note that V_O1_ and V_O2_ are symmetrically equivalent in the crystal structure of TiO_2_. (**b**) The possible O vacancy sites V_O3_, V_O4_, V_O5_, and V_O6_ of the Ce-doped TiO_2_. Vacancies sites V_O3_, V_O4_ are in a [CeO_6_] octahedron, whereas V_O5_, and V_O6_ sites are in the next-nearest-neighboring [TiO_6_] octahedron. The large blue balls, large green balls, and small red balls represent the Ti, Ce, and O atoms, respectively. The [TiO_6_] and [CeO_6_] octahedrons are shown in blue and green, respectively. This figure was drawn by using the VESTA 3 software^20^. https://jp-minerals.org/vesta/en/download.html.

**Figure 7:**
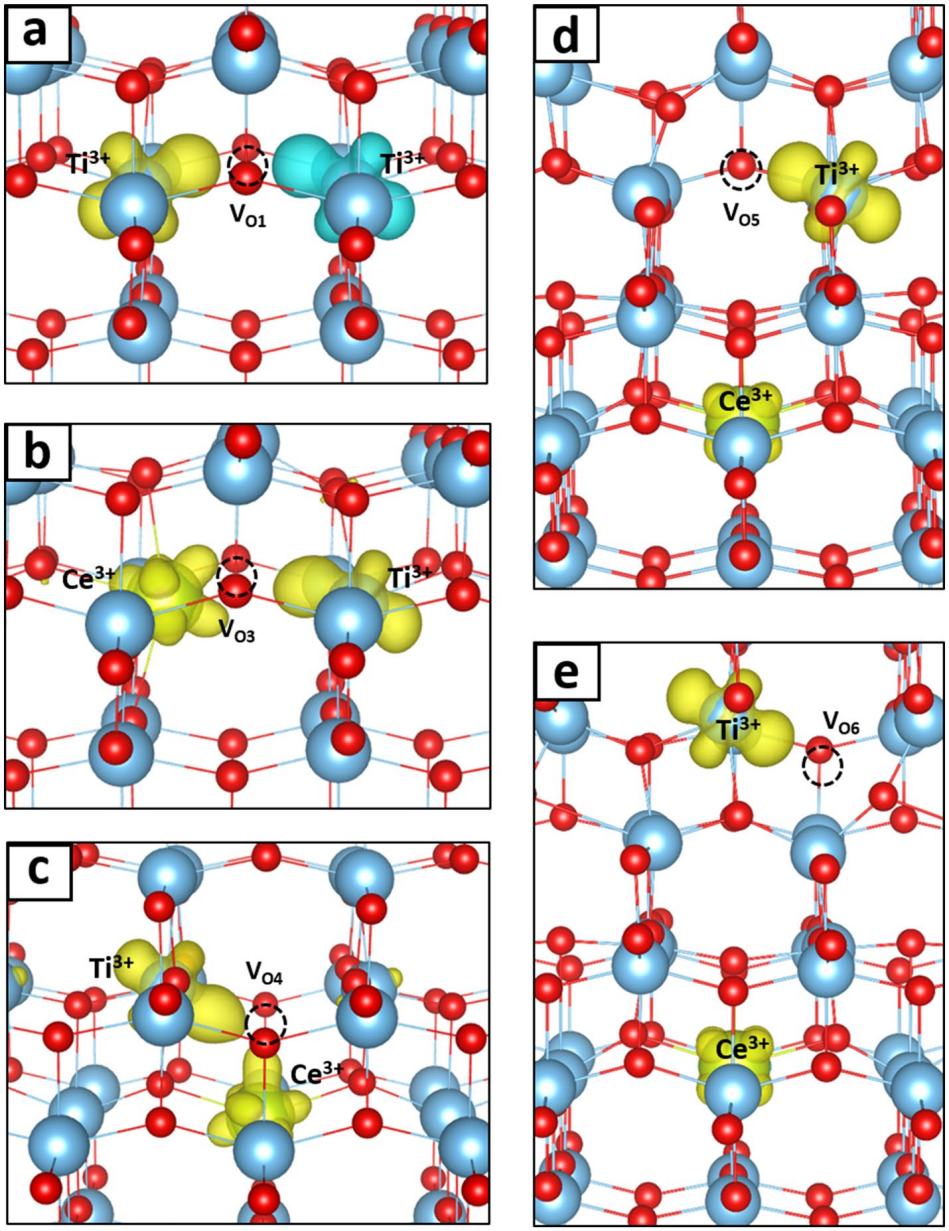
Structure and spin density isosurface of (**a**) oxygen vacancy V_O1_, (**b**) oxygen vacancy V_O3_, (**c**) oxygen vacancy V_O4_, (**d**) oxygen vacancy V_O5_, and (**e**) oxygen vacancy V_O6_. The α and β spin densities are shown in yellow and blue, respectively. See text for a more detailed description of the different structures. Isosurface: 0.006 e^−^/Å^3^. This figure was drawn by using the VESTA 3 software^20^. https://jp-minerals.org/vesta/en/download.html.

Oxygen vacancy defects on different sites in TiO_2_ and Ce-doped TiO_2_ are also considered in the structural model. As shown in Fig. 6a, an O atom was removed from either the O1 or O2 site of the [TiO_6_] octahedron in the undoped TiO_2_ model. We note that the O1 and O2 sites are symmetrically equivalent in the crystal structure of TiO_2_. On the other hand, one of the O vacancies on the O3 and O4 sites of the [CeO_6_] octahedron and the O5 and O6 sites of the [TiO_6_] octahedron was created for each defective Ce-doped TiO_2_ model as shown in Fig. 6b. Geometry optimization was carried out for all structural models with a 5 × 5 × 3 Monkhorst–Pack k-point grid. The atomic coordinates and the lattice parameters were optimized until the maximum force on each atom was less than 0.02 eV/Å with an electronic self-consistency convergence of 10^−6^ eV. The spin density isosurfaces are shown in Fig. 7, corresponding to the trapped electrons that lead to the defect levels in the energy gap. While the electron spin densities for defect states near oxygen vacancies V_O1_ in pure TiO_2_ are in antiferromagnetic orientation, they are in ferromagnetic orientations near oxygen vacancies V_O3_, V_O4_, V_O5_, and V_O6_ in Ce-doped TiO_2_, as indicated in Fig. 7. Therefore, if oxygen vacancies can be created by UV irradiation in the Ce-doped TiO_2_ samples, the ferromagnetically oriented spin densities near such oxygen vacancies are likely responsible for the experimentally observed enhancement of magnetism in the UV irradiated samples of Ce-doped TiO_2_.

To elucidate the possible mechanism for creation of oxygen vacancies by UV irradiation in the pure and Ce-doped samples of TiO_2_, the formation energies of O vacancy ${E}_{VO}^{F}$ were obtained as the difference in total energies between two supercells using the following equation:

$${E}_{VO}^{F}\left(q\right)={E}_{T}\left({Ce}_{n}{Ti}_{32-n}{O}_{63},q\right)-{E}_{T}\left({Ce}_{n}{Ti}_{32-n}{O}_{64},q\right)+\frac{1}{2}{E}_{T}({O}_{2})$$

where ${E}_{T}\left({Ce}_{n}{Ti}_{32-n}{O}_{63},q\right)$ and ${E}_{T}\left({Ce}_{n}{Ti}_{32-n}{O}_{64},q\right)$ are the total energies of the optimized supercells with and without an O vacancy, respectively, $q$ is the number of electrons removed from the supercell by UV irradiation, and ${E}_{T}\left({O}_{2}\right)$ is the total energy for the ground triplet state of an optimized oxygen molecule in the gas phase.

The oxygen vacancy formation energies in all defect structures discussed above are summarized in Supplementary Table S3 online. In the pure TiO_2_ model, although the Ti–O_1_ and Ti–O_2_ bonds in a [TiO_6_] octahedron have different bond lengths, the two O atoms O_1_ and O_2_ are symmetrically equivalent in the crystal structure and therefore the O vacancy formation energy for both sites have the same value of 4.98 eV. As shown in Fig. 7a, when an O atom is removed from TiO_2_, the two electrons left in the O vacancy are transferred to the two nearest Ti atoms, forming Ti^3+^ defect states in an antiferromagnetic orientation. In the presence of a valence hole created by UV-light irradiation, only one electron is transferred and the Coulomb energy of the system is thus largely lowered. The O vacancy formation energy is therefore lowered to 2.51 eV. When there are two valence holes, the O vacancy formation energy is further lowered to 0.43 eV.

In the Ce-doped TiO_2_ model, with a Ce atom on the octahedral Ti site, the O atoms surrounding Ce in the [CeO_6_] octahedron are not symmetrically equivalent. Therefore, the O vacancies at the O3 and O4 sites of the [CeO_6_] octahedron are considered separately. For O atom removal from the O3 site and the O4 site shown in Fig. 7b,c, respectively, the two electrons left in the O vacancy will be transferred to the Ce atom and the nearest Ti atom, forming Ce 4f and Ti 3*d* defect states in a ferromagnetic orientation for both cases. In comparison to the V_O3_ structure, only a small structural relaxation is observed in the V_O4_ structure, leading to a higher O vacancy formation energy of 4.64 eV at the O4 site than that of 4.08 eV at the O3 site. Therefore, the V_O3_ structure is energetically favored compared to the V_O4_ structure. In the presence of a valence hole generated by UV-light irradiation, only one electron is left in the Ce 4f state. The O vacancy formation energies at the O3 and O4 sites decreased to 0.95 eV and 2.30 eV, respectively. When two valence holes are created, their O vacancy formation energies are further lowered to − 0.74 eV and 0.42 eV, representing the onset of an O vacancy formation at the O3 site. We therefore have theoretically demonstrated the Ce reduction mechanism induced by valence–hole-generating UV-light irradiation. Finally, when an oxygen atom is removed from the O5 or O6 sites, the two electrons left will be transferred to the nearest Ti atom and the Ce atom, forming Ti^3+^ and Ce^3+^ defect states in ferromagnetic orientations as demonstrated in Fig. 7d,e. The formation energies of O5, O6 oxygen vacancies were calculated to be 4.86 eV and 4.82 eV, respectively. As the UV irradiation generated one and two valence holes, the formation energies were reduced to 2.56 eV and 0.51 eV for the O5 vacancy and 2.57 eV and 0.51 eV for the O6 vacancy, respectively. It is worth noting that the formation of the oxygen vacancies in the sample is followed by the relaxation of the UV-generated valence holes, leaving the Ce dopant ions in their reduced valence state as is incorporated into the magnetic calculations above.

To summarize the theoretical results, we note that the formation of oxygen vacancies at the O3 sites in the supercells of the Ce-doped TiO_2_ structure is energetically favorable and thus tends to occur when two electrons are removed from each supercell of the crystal by UV irradiation. Our calculations also indicate that the electron spin densities for defect states near V_O3_ are in ferromagnetic orientations. Therefore, we have theoretically demonstrated that UV irradiation can generate oxygen vacancies near Ce dopant atoms with electron spin densities in ferromagnetic orientations, leading to the experimentally observed reduction of Ce and enhancement of magnetic properties in Ce-doped TiO_2_.

## Conclusion

We experimentally observed generation of oxygen vacancy defects and consequent reduction of Ce in Ce-doped TiO_2_ diluted magnetic semiconductor samples as a result of UV-light irradiation by using synchrotron radiation X-ray techniques including XANES and EXAFS. The variation of electronic states observed by UV–Vis diffuse reflectance and photoluminescence measurements also shows reduction of Ce and oxygen vacancy generation in the samples due to UV irradiation. The increase of oxygen vacancies led to substantially enhanced room-temperature ferromagnetism as demonstrated by our SQUID results. The formation of oxygen vacancy defects and enhancement of magnetism were also theoretically explained by our first-principle DFT calculations. Cerium-doped TiO_2_ diluted magnetic semiconductors with room-temperature ferromagnetism enhanced by UV-light irradiation are potentially useful for many technological applications.

## Methods

Nanocrystal samples of Ce-doped TiO_2_ were prepared using a sol–gel method. The crystal structures and particle sizes of the samples were obtained from synchrotron-based XRD using the Scherrer equation. Coupled plasma mass spectrometry (ICPMS) measurements were employed to determine the Ce concentration of the samples. The Ce-doped TiO_2_ samples were then irradiated by UV-light from a UVC lamp (6 W, UVGL-58, UVP) with various irradiation time durations in ambient conditions. The Ce L_3_-edge and Ti K-edge XANES and EXAFS, performed at beamline BL07A of the Taiwan Light Source (TLS) at National Synchrotron Radiation Research Center (NSRRC), were used to reveal the evolution of valances and local structures surrounding Ce and Ti atoms as a result of UV irradiation, respectively. Electronic structural changes due to UV-light irradiation were monitored by UV–Vis diffuse reflectance measurements using a spectrophotometer (UV-4150, Hitachi). Photoluminescence (PL) spectra were also measured using an excitation source of wavelength 325 nm to investigate the variation of defect states due to UV-light irradiation. The UV-induced magnetic-property changes were exhibited by the M–H and M–T data obtained by using a superconducting quantum interference device (SQUID). Finally, the experimentally observed effects of UV irradiation on Ce-doped TiO_2_ were theoretically explained by first-principle DFT calculations using the VASP code.

## Supplementary Information

Supplementary Information.

## Data Availability

All data generated or analyzed during this study are included in the main text and supplementary information of this published article.
