# Supplementary material for: Reduction of dopant ions and enhancement of magnetic properties by UV irradiation in Ce-doped TiO2
Source: Sci Rep. 2021 Apr 7;11:7668. doi: 10.1038/s41598-021-87115-z (PMC8027894; doi:10.1038/s41598-021-87115-z)
Supplement: Supplementary file 1 — Supplementary Information. [file 41598_2021_87115_MOESM1_ESM.docx]

**Reduction of dopant ions and enhancement of magnetic properties by UV irradiation in Ce-doped TiO_2_**

Tai-Sing Wu^1^, Leng-You Syu^2^, Bi-Hsuan Lin^1^, Shih-Chang Weng^1^, Horng-Tay Jeng^2,3^, Yu-Shan Huang^1^, and Yun-Liang Soo*^1,2^

*^1^ National Synchrotron Radiation Research Center, Hsinchu, Taiwan*

*^2^ Department of Physics, National Tsing Hua University, Hsinchu, Taiwan*

*^3^Institute of Physics, Academia Sinica, Taipei, Taiwan*

**Supplementary Information**


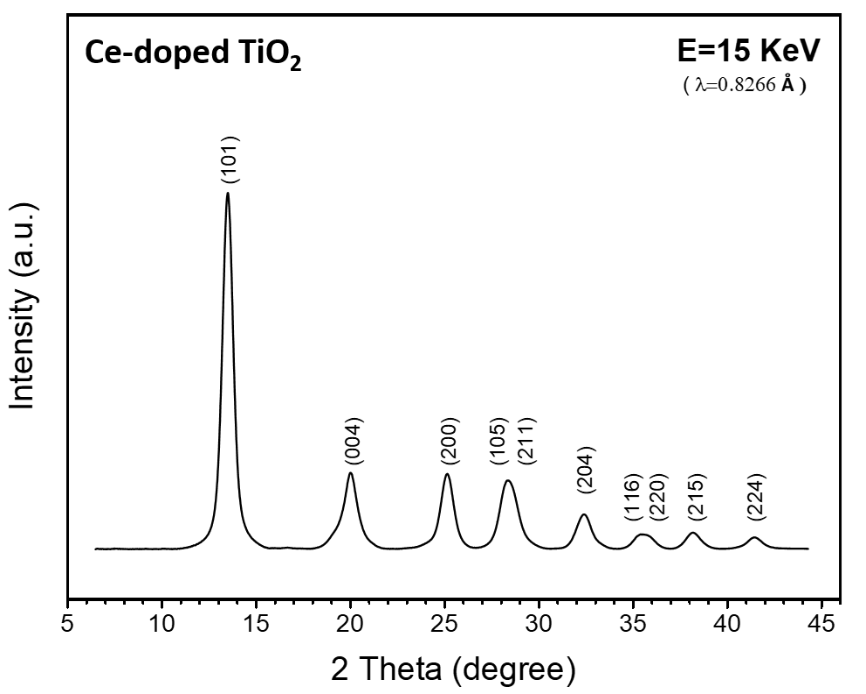


Supplementary Figure S1 – The XRD pattern of as-prepared Ce-doped TiO_2_ nanocrystal sample.

The variation of defect states due to UV-light irradiation can also be revealed by photoluminescence measurements. As shown in Supplementary Figure S2 on line, an emission peak associated with F^+^ center at around 550 nm was observed in the PL spectrum for the as-made sample. After UV-light irradiation, the peak intensity increased and an additional peak ascribed to Ti^3+^/F centers emerged at 450 nm. The increase of intensity for the peak at 550 nm and the appearance of the new peak at 450 nm may have resulted from substantial increase of oxygen vacancy concentration due to UV irradiation, revealed in the x-ray analysis.


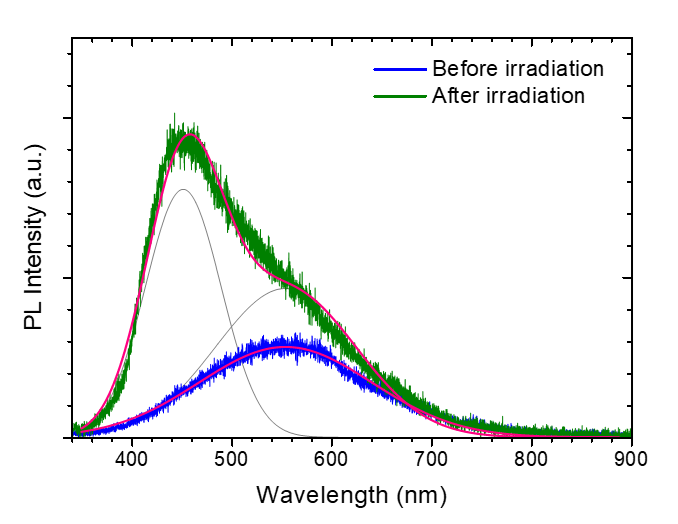


Supplementary Figure S2– Photoluminescence spectra of Ce-doped TiO_2_ sample before and after 600 minutes of UV-light irradiation.

**Supplementary Table S1.** Parameters of local structure around Ce atoms obtained from curve-fitting of the Ce L_3_-edge EXAFS for sample before and after 600 minutes of UV-light irradiation. N is the coordination number. R is the bond length. σ^2^ is the Debye-Waller-like factor serving as a measure of local disorder. ΔE_0_ is the difference between the zero kinetic energy value of the sample and that of the theoretical model used in FEFF.

| **Sample** | **Bond** | ***N*** | ***R***  **(Å)** | **σ^2^**  **(10^-3^ Å^2^)** | **Δ*E_0_***  **(eV)** |
| --- | --- | --- | --- | --- | --- |
| As-made | Ce-O | 5.7 ± 0.5 | 2.13 ± 0.01 | 6.9 ± 1.1 | -1.5 ± 0.8 |
|  | Ce-Ti | 3.4 ± 0.3 | 3.18 ± 0.02 | 7.6 ± 1.0 | -5.1 ± 0.8 |
| 600 minutes | Ce-O | 3.8 ± 0.3 | 2.13 ± 0.01 | 5.4 ± 1.0 | -2.9 ± 0.9 |
|  | Ce-Ti | 3.5 ± 0.4 | 3.22 ± 0.02 | 12.0 ± 1.3 | -3.1 ± 0.8 |

**Supplementary Table S2.** Parameters of local structure around Ti atoms obtained from curve-fitting of the Ti K-edge EXAFS for sample before and after 600 minutes of UV-light irradiation.

| **Sample** | **Bond** | ***N*** | ***R***  **(Å)** | **σ^2^**  **(10^-3^ Å^2^)** | **Δ*E_0_***  **(eV)** |
| --- | --- | --- | --- | --- | --- |
| As-made | O | 1.9 ± 0.3 | 1.95 ± 0.01 | 2.2 ± 1.4 | -0.6 ± 1.8 |
|  | O | 0.9 ± 0.2 | 1.97 ± 0.01 | 2.2 ± 1.4 | -0.6 ± 1.8 |
|  | Ti | 1.5 ± 0.4 | 3.07 ± 0.01 | 1.6 ± 1.3 | 1.1 ± 2.3 |
|  | Ti | 2.2 ± 0.6 | 3.82 ± 0.02 | 1.6 ± 1.3 | 1.1 ± 2.3 |
|  | O | 6.1 ± 1.4 | 3.93 ± 0.02 | 1.6 ± 1.3 | -0.6 ± 1.8 |
| 600 minutes | O | 1.7 ± 0.3 | 1.96 ± 0.01 | 2.0 ± 1.0 | 1.1 ± 2.6 |
|  | O | 0.9 ± 0.2 | 1.98 ± 0.01 | 2.0 ± 1.0 | 1.1 ± 2.6 |
|  | Ti | 1.3 ± 0.4 | 3.05 ± 0.01 | 2.1 ± 1.5 | -2.1 ± 1.4 |
|  | Ti | 1.7 ± 0.4 | 3.81 ± 0.02 | 2.1 ± 1.5 | -2.1 ± 1.4 |
|  | O | 5.2 ± 1.1 | 3.94 ± 0.02 | 2.1 ± 1.5 | 1.1 ± 2.6 |

**Supplementary Table S3.** Formation energy (eV) of O vacancy on different sites with various numbers of electrons removed.

| **number of electrons removed** | | **0** | **1** | **2** |
| --- | --- | --- | --- | --- |
| **TiO_2_** | **V_O1_** | 4.98 | 2.51 | 0.43 |
|  | **V_O2_** | -- | -- | -- |
| **Ce-doped TiO_2_** | **V_O3_** | 4.08 | 0.95 | -0.74 |
|  | **V_O4_** | 4.64 | 2.30 | 0.42 |
|  | **V_O5_** | 4.86 | 2.56 | 0.51 |
|  | **V_O6_** | 4.82 | 2.57 | 0.51 |
